# Numerical Simulation and Optimization of Drug-Coated Balloon Inflation for Vascular Stenosis

**DOI:** 10.3390/bioengineering13030301

**Published:** 2026-03-05

**Authors:** Chenzhao Zhang, Yuanyuan Zhang, Shengzhang Wang

**Affiliations:** 1 College of Biomedical Engineering, Fudan University, Shanghai 200433, China; 2 Yiwu Research Institute, Fudan University, Yiwu 322000, China

**Keywords:** drug-coated balloon, vascular stenosis, numerical simulation, finite element analysis, paclitaxel

## Abstract

This study establishes a numerical model for the implantation of drug-coated balloons (DCBs) in blood vessels, in order to quantify drug (paclitaxel) transfer and diffusion within stenotic vessels and provide a reference for clinical surgical plans. Objective: To study the change in paclitaxel concentration over time in the blood vessel after the implantation of a DCB in vessels with different degrees of stenosis, and thereby determine the optimal balloon dilation time. Method: Using finite element modeling and numerical simulation techniques, a model was established to study the rules of paclitaxel concentration change over time in vessels with different degrees of stenosis. Results: Based on the simulation prediction, the longer the balloon dilation time during implantation, the more paclitaxel enters the vessel wall. After the balloon is withdrawn, the paclitaxel gradually diffuses evenly throughout the vessel, and the paclitaxel concentration gradually decreases over time. Conclusion: Under the simulation conditions, the optimal balloon dilation times for vessels with stenosis rates of 10%, 30%, and 50% should be 20 s, 60 s, and 80 s, corresponding effective duration of 6 weeks, 6 weeks and 4 weeks, respectively.

## 1. Introduction

Vascular stenosis is a significant cause of life-threatening health issues worldwide, with a history of human struggle against it for nearly a century [1]. Cardiovascular diseases account for approximately 30% of global mortality, and remain the leading cause of death in China, contributing to over 40% of all-cause mortality in urban and rural populations [2,3,4]. Traditionally, diseases caused by vascular stenosis, such as coronary heart disease and ischemic stroke, are usually treated by open-chest or open-skull surgeries. These procedures carry high risks and require a long recovery time, undoubtedly bringing heavy physical and psychological burdens to the patients. The advent of interventional treatment methods has changed this situation.

The development of interventional treatment techniques has gone through stages from balloon angioplasty, bare metal stents to drug-eluting stents [5,6,7,8,9]. However, permanent metal stents like stainless steel and cobalt–chromium alloy stents can cause inflammatory reactions due to their long-term presence in the body, leading to a series of adverse consequences such as intimal hyperplasia and late-stage stent thrombosis [10,11,12].

Drug-coated balloons (DCBs) have emerged in recent years as a treatment method [13,14,15,16]. They have achieved good therapeutic effects in the treatment of many vascular diseases, such as native vessel stenosis, in-stent re-stenosis, and small vessel diseases [17], hence gaining considerable attention in the medical and academic fields. Essentially, a DCB is a targeted drug delivery device for diseased vessels. The amount of drug absorbed by the vessel during a single implantation and the change in drug concentration in the vessel over time after implantation determine the therapeutic effect of the surgery. Computational simulation is a good and effective way to study the drug diffusion process in the vessel [18,19]. The relationship between balloon dilation time and drug absorption, as well as the drug diffusion process within the blood vessels over time, are crucial for improving the effectiveness and safety of DCB treatment. However, systematic studies on this aspect are still scarce [20,21,22,23].

Therefore, based on the previous in vitro implantation experiments of DCBs [23,24], which focused on the experimental results of paclitaxel transfer in in vitro vessels, this study establishes a numerical simulation model of the drug transfer process from the balloon surface to the vessel, and studies the diffusion rule of the drug over time after the balloon is implanted in vessels with different degrees of stenosis. We combine the effectiveness and safety standards for the clinical use of DCBs in treating vascular stenosis to determine the optimal balloon dilation time for vessels with different degrees of stenosis, which aims to maximize the time duration with effective concentration while ensuring that the concentration is lower than the toxic criteria.

## 2. Materials and Methods

In the procedure of DCB implantation, once the balloon reaches the diseased site, pressure is applied to inflate the balloon, which in turn dilates the stenotic blood vessel. To more accurately simulate the actual surgical process, this study carried out a finite element analysis of the balloon dilation process within the vessel. The obtained results, namely the inflated balloon and vessel models, were imported into Ansys Fluent 2020 (V2020, Ansys Corporation, Canonsburg, PA, USA) to perform numerical simulations of the paclitaxel transfer process from the balloon surface to the vessel wall. The paclitaxel transfer process from the balloon surface to the inner vessel wall can be divided into two parts: numerical simulation of the ex vivo testing vessel and numerical simulation of vessels with different stenosis rates. First, a numerical simulation of the ex vivo test vessel is performed and compared with the in vitro experimental results to determine the transfer coefficient of the paclitaxel transfer process from the balloon surface to the inner vessel wall. Then, the obtained transfer coefficient is assigned to the stenosis vessel model for numerical simulation, and the drug content is successfully transferred to the inner vessel walls of three different stenosis rates immediately after balloon withdrawal can be obtained.

After the drug transfers from the balloon surface to the inner vessel wall, its diffusion process does not end. Over time, the drug continues to diffuse towards the vessel’s tunica media, tunica externa and both ends. Therefore, based on the numerical simulation results of the drug transfer process from the balloon surface to the stenotic vessel, after calculating the paclitaxel content transferred to the inner vessel wall after different balloon dilation times, we use it as the initial condition for the numerical simulation of free paclitaxel diffusion in the vessel over time. This can result in the pattern of paclitaxel concentration changes over 8 weeks in vessels with three stenosis rates after balloon withdrawal. Finally, the obtained numerical simulation results combined with the standard for clinical use of drug balloons determine the optimal balloon dilation time for vessels with different stenosis rates.

### 2.1. Establishment of the Model of DCB Dilation in Vessels

#### 2.1.1. Geometric Model and Grid Partitioning

**Figure 1:**
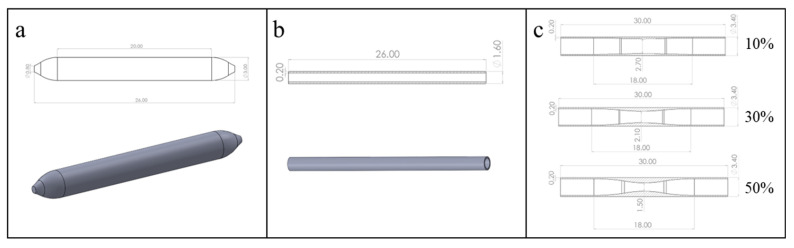
Geometric models of the DCB and blood vessels: (**a**) DCB model; (**b**) the ex vivo vessel model; (**c**) vessel models with different stenosis rates (10%, 30%, 50%).

Based on in vitro DCB implantation experiments, we first established the geometric models of the drug balloon, as shown in Figure 1a, and the ex vivo vessel, as shown in Figure 1b [24]. In addition, considering the commonly observed stenosis rates in clinical practice, we established geometric vessel models with stenosis rates of 10%, 30%, and 50%, as shown in Figure 1c. Next, we used Hypermesh 2019 software (V2019, Altair Corporation, Fremont, CA, USA) for grid generation. The vessel was divided into hexahedral elements, and the grid size was set to 0.02 mm. C3D8R elements were used for simulation; the balloon was divided into triangular elements, considered shell elements in the calculation, and set to a uniform thickness of 0.10 mm. Finally, the balloon and vessel models with divided grids were imported into ABAQUS 2018 software (V2018, Dassault Corporation, Vélizy-Villacoublay, France) for finite element analysis of the balloon dilation process.

#### 2.1.2. Boundary Conditions and Material Parameter Settings

Since the dilation of the balloon has a relatively small impact on the axial length of the vessel wall, the boundary conditions of the vessel are set to allow radial dilation at both ends, i.e., no displacement in the axial direction; the boundary conditions of the balloon are completely fixed at both ends.

An isotropic Ogden constitutive model, as written in Equation (1), was adopted as a reasonable approximation for the overall vessel mechanical response for the vessel wall [25], where ${\bar{\lambda }}_{i}=J^{-\frac{1}{3}}\lambda_{i}$, ${\bar{\lambda }}_{i}$ is the reduced principal elongation and $\lambda_{i}$ is the principal elongation. $\mu_{i}$, $\alpha_{i}$ and $D_{i}$ are temperature-dependent material parameters. We used a linear elastic constitutive model for the medical nylon balloon [26]. The material properties of the vessel wall and balloon are shown in Table 1 and Table 2, respectively.

(1) $$U=\sum_{i=1}^{3}\frac{2\mu_{i}}{\alpha_{i}^{2}}\left({\bar{\lambda }}_{1}^{\alpha_{i}}+{\bar{\lambda }}_{2}^{\alpha_{i}}+{\bar{\lambda }}_{3}^{\alpha_{i}}-3\right)+\sum_{i=1}^{3}\frac{1}{D_{i}}{\left(J^{el}-1\right)}^{2i}$$

#### 2.1.3. Model of the DCB After Dilation in the Vessel

**Figure 2:**
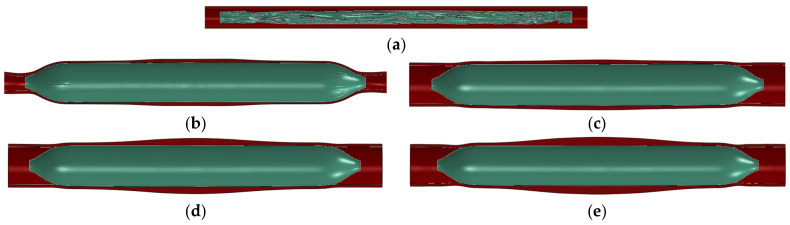
Comparison before and after balloon dilation: (**a**) before balloon dilation in the ex vivo vessel; (**b**) after balloon dilation (ex vivo vessel); (**c**) after balloon dilation (vessel stenosis rate of 10%); (**d**) after balloon dilation (vessel stenosis rate of 30%); (**e**) after balloon dilation (vessel stenosis rate of 50%).

Figure 2 shows a comparison between before and after balloon dilation in the vessel; the green represents the balloon, and the red area represents the vessel wall. It can be observed that the vessel has deformed after balloon dilation. After the finite element analysis process is completed, the balloon and vessel models in the inflated state are obtained.

### 2.2. Numerical Simulation of the Paclitaxel Transfer Process

The transfer of the paclitaxel from the balloon surface to the vessel can be understood as a short-term diffusion process, which can be simulated by solving the diffusion equation in the balloon domain and the vessel wall. In Ansys Fluent 2020, the concentration convection diffusion equation can be solved in the solution domain by setting a user-defined scalar (UDS).

The expression for the convection diffusion equation is

(2) $$\frac{\partial \phi }{\partial t}+\frac{\partial }{\partial x_{i}}\left(u_{i}\phi -\Gamma \frac{\partial \phi }{\partial x_{i}}\right)=S_{\phi },i=1,2,3,$$

where we define the UDS ϕ as the concentration (unit: kg/m^3^), u_i_ represents the velocity in three directions, x_i_ represents the coordinates in three directions, and S_ϕ_ is the source term of the convection diffusion equation. Γ is the transfer coefficient of the drug from the balloon to the vessel.

The process of drug transfer from the balloon to the vessel wall has no convection so $\frac{\partial u_{i}\phi }{\partial x_{i}}\;$= 0. In addition, this study does not consider the absorption of the drug by cells during the diffusion process, so the source term $S_{\phi }$ = 0. The final diffusion equation is

(3) $$\frac{\partial \phi }{\partial t}-\frac{\partial }{\partial x_{i}}\left(\Gamma \frac{\partial \phi }{\partial x_{i}}\right)=0,i=1,2,3$$

#### 2.2.1. Establishment of a Numerical Simulation Model for the Transfer Process of Drugs

**Figure 3:**
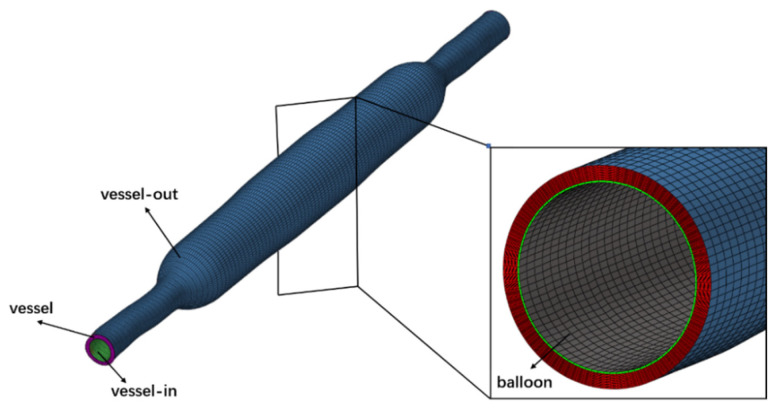
Mesh diagram of ex vivo experiment vessel and balloon after dilation.

The meshes of the experimental vessel and balloon model obtained from the finite element analysis of the DCB dilation in the vessel are extracted and the solid meshes of the vessel and balloon are created in Hypermesh 2019, followed by naming the boundaries separately. Based on the mesh independence test of vessel drug concentration in the time point of 24 h after balloon dilation, the final mesh size of 0.01 mm was used for the vessel and balloon in this numerical model. Figure 3 is a schematic diagram of the mesh of the numerical model of l vessel and balloon after dilation.

Since the balloon’s contact time with the vessel during dilation is relatively short, it is assumed that no drug is lost from the vessel surface and balloon surface during the dilation of the DCB, so all four surfaces are set to the second type of boundary conditions (i.e., flux conditions), with a flux of zero. The initial concentration value of the balloon mesh cell is set to 75.32 kg/m^3^ based on the experimental results of the drug mass carried by the balloon before implantation, serving as the initial conditions for the simulation [15].

#### 2.2.2. Determination of the Transfer Coefficient

The determination of the transfer coefficient in the process of drug transfer from the surface of the balloon to the inner wall of the vessel during balloon dilation can be achieved by setting a series of different transfer coefficient values for the numerical simulation model of the experimental vessel and balloon. Then, the numerical simulation results of the drug transfer process from the surface of the balloon to the inner wall of the ex vivo experimental vessel are compared with the in vitro experimental results in the literature [15]. When the total error between the numerical simulation results and the experimental results at different expansion times is less than 0.5%, it is considered that the set transfer coefficient is the actual transfer coefficient.

### 2.3. Numerical Simulation of the Drug Transfer Process from the Balloon Surface to the Inner Wall of the Narrowed Vessel

#### 2.3.1. Setting up the Computational Model

**Figure 4:**
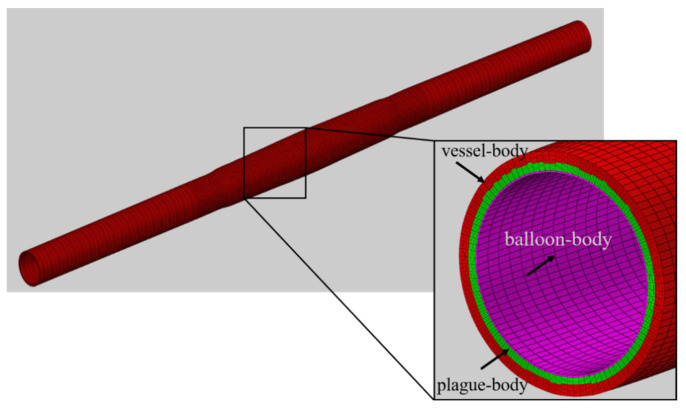
Mesh diagram of stenotic vessel and balloon after dilation (stenosis rate is 50%).

The naming of the vessel, vessel-in, vessel-out, and balloon for the dilation model of vessels with different stenosis rates and balloon adopts the same method as in Section 2.2.1. Figure 4 shows the mesh diagram of the model after the dilation of a vessel with a 50% stenosis rate, where vessel–body, balloon–body, and plaque–body represent the body meshes of the vessel domain, balloon domain, and plaque domain, respectively. Furthermore, plaque–balloon, vessel–balloon, and vessel–plaque are defined to represent the inner surfaces between the plaque and balloon, vessel and balloon, and vessel and plaque, respectively.

#### 2.3.2. Setting up Boundary Conditions and Initial Conditions

The vessel surface, vessel-in surface, vessel-out surface, and balloon surface are also set to the second type of boundary conditions (i.e., flux conditions), with a flux of zero; the boundary conditions of plaque–balloon surface, vessel–balloon surface, and vessel–plaque surface are set as coupled boundary conditions, indicating that the drug can freely pass through these surfaces. The initial value of the balloon mesh cell remains at 75.32 kg/m^3^ [15] as the initial condition for the simulation calculation. The transfer coefficient in the process of drug transfer from the balloon surface to the vessel inner wall is set as the value fitted from the numerical simulation and experimental results of the ex vivo vessel.

### 2.4. Numerical Simulation of the Free Diffusion Process of the Drug in the Vessel over Time After the Balloon Is Withdrawn

Since the balloon has been withdrawn when the drug freely diffuses in the vessel, the balloon domain (balloon–body in Figure 4) in the model is removed, and the balloon surface in the model will be automatically deleted at this time.

During the free diffusion of the drug in the diseased vessel, the loss from the vessel surface, vessel-in surface, and vessel-out surface is not considered, so these three surfaces are set to the second type of boundary condition (i.e., flux boundary conditions), with a value of zero.

The vessel–plaque surface does not participate in the calculation during the free diffusion process of the drug, so the boundary conditions are still set as coupled boundary conditions.

Setting up boundary conditions for the plaque–balloon surface and vessel–balloon surface is as follows. After the balloon is withdrawn, the continuous flushing of the vessel inner wall by the blood flow will cause the drug to continuously lose from the plaque–balloon surface and vessel–balloon surface, thereby causing the total amount of drug in the vessel to gradually decrease. Since the contact area of the plaque–balloon surface is much larger than that of the vessel–balloon surface, it can be assumed that all the drugs are lost from the plaque–balloon surface. Therefore, according to the in vivo experimental results of Liu et al [27]., the boundary conditions of the plaque–balloon surface can be set as follows: about 70% of the total mass of the drug in the vessel will be lost in the first 24 h after the balloon is withdrawn, the decline rate slows down after 24 h, and the loss amount each day afterwards is about 30% of the total mass of the previous day [27]. The boundary conditions of the vessel–balloon surface are set as the second type of boundary condition (flux conditions), with a value of 0. The diffusion coefficient of paclitaxel in vessels and plaques is set to 1 × 10^−12^ m^2^/s [28].

## 3. Results

### 3.1. Numerical Simulation Results of Paclitaxel Transfer from the Balloon Surface to the Ex Vivo Vessel and Determination of Transfer Coefficient

Table 3 shows the total error between the numerical simulation results and experimental results from our previous study [24]. The total error is defined as the sum of the relative errors between the simulated and experimental values at two dilation timepoints (60 s and 120 s). According to the data in the table, when the transfer coefficient is set to 1.28 × 10^−12^ m^2^/s, the total error between the numerical simulation results and experimental results under different dilation times is less than 5%. Therefore, the transfer coefficient is determined to be 1.28 × 10^−12^ m^2^/s.

### 3.2. Numerical Simulation Results of Paclitaxel Transfer from the Balloon Surface to the Narrowed Vessel

**Figure 5:**
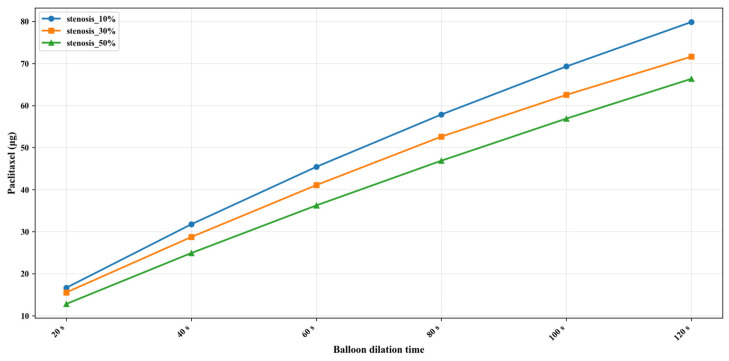
Paclitaxel distribution (μg) in the 50% stenosis rate vessel model under 120 s dilation time.

**Figure 6:**
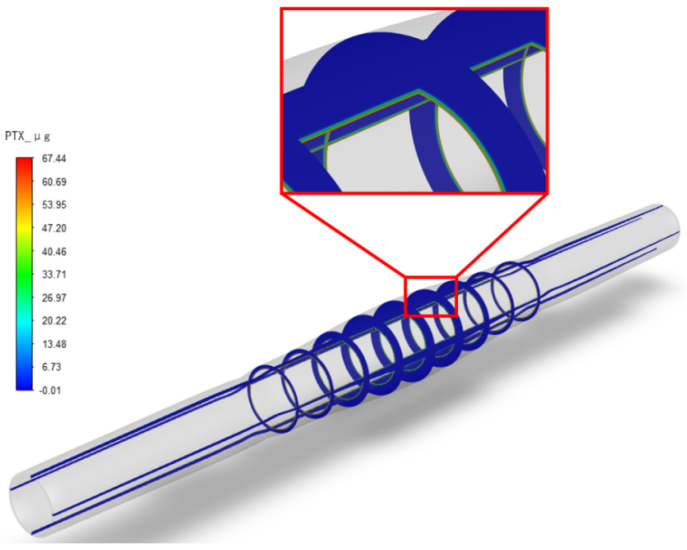
Amount of paclitaxel in the blood vessel after different balloon dilation times (μg).

After setting up the three different stenosis rate vessel models, the paclitaxel content transferred to the vessel at different dilation times (unit: μg) was obtained, as shown in Figure 5. The initial paclitaxel concentration is lower in vessels with a higher stenosis rate, which may be caused by the reduction in balloon contact area because of large plaque. Figure 6 shows the paclitaxel distribution in the 50% stenosis rate vessel model when the balloon is inflated for 120 s. It can be seen that the drugs transferred from the balloon to the vessel mainly concentrate on the inner wall of the vessel in contact with the balloon during the balloon dilation process.

### 3.3. Numerical Simulation of the Free Diffusion Process of the Paclitaxel

**Figure 7:**
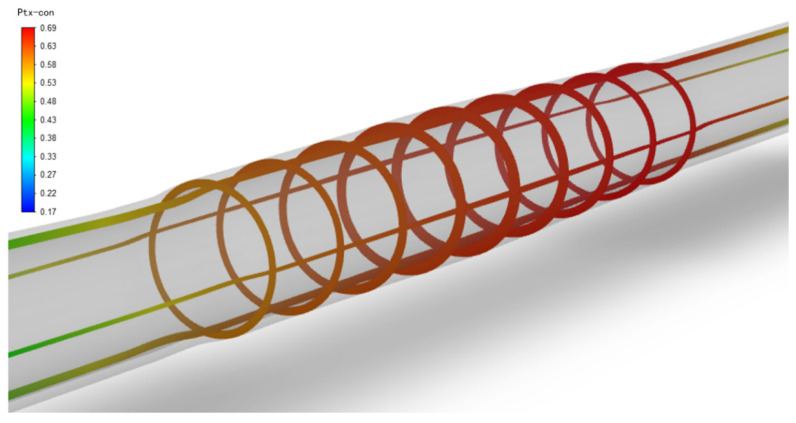
Paclitaxel concentration distribution in a 50% stenosis rate vessel (μg/g), balloon dilation time of 120 s, and 5 weeks after balloon withdrawal.

After the balloon is withdrawn and the paclitaxel diffuses in the vessels with three different stenosis rates for some time, the paclitaxel originally concentrated on the inner wall of the vessel will gradually diffuse evenly. Figure 7 shows the paclitaxel distribution in the vessel with a 50% stenosis rate, a balloon dilation time of 120 s, and at the 5th week after the balloon is withdrawn. The paclitaxel concentration in most areas is above the effective concentration, with a maximum concentration of 0.69 μg/g on the inner surface of the stenosis area.

The current clinical safety and effectiveness criteria for DCBs are as follows: on the one hand, within 24 h after the balloon is implanted, the paclitaxel concentration in the vessel should be reduced to below the toxic concentration (85.40 μg/g) [29]; on the other hand, the paclitaxel concentration in the vessel should be maintained above the effective concentration (0.047 μg/g) [29] for as long as possible (within 2 months) to reduce the time the patient takes antiplatelet drugs and achieve the best overall treatment effect. Therefore, by combining the numerical simulation results obtained in this section with clinical criteria, the optimal balloon dilation time for vessels with different stenosis rates can be determined.

**Figure 8:**
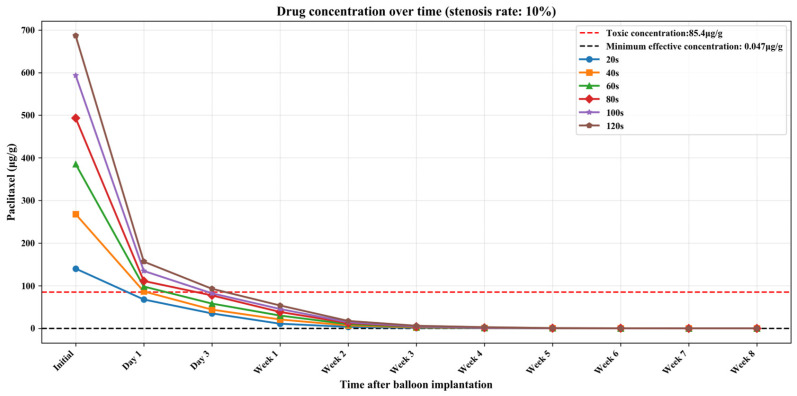
The curve of paclitaxel concentration over time at different balloon dilation times (stenosis rate is 10%).

**Figure 9:**
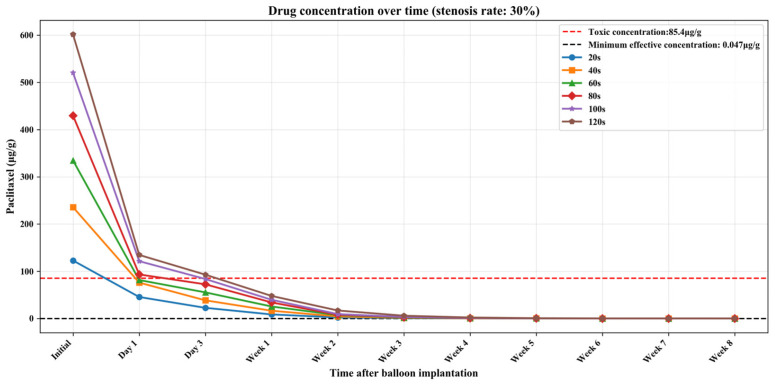
The curve of paclitaxel concentration over time at different balloon dilation times (stenosis rate is 30%).

**Figure 10:**
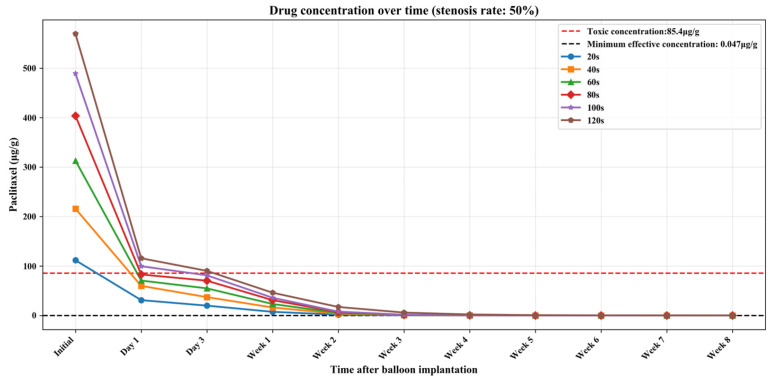
The curve of paclitaxel concentration over time at different balloon dilation times (stenosis rate is 50%).

Tables S1–S3 (Supplementary Materials) show the numerical simulation results of drug concentration changes over time under different balloon dilation times in vessels with stenosis rates of 10%, 30%, and 50%, respectively. In addition, Figure 8, Figure 9 and Figure 10 show that: (1) in vessels with the same stenosis rate, if the balloon dilation time is longer, the initial drug concentration in the vessel after the balloon is withdrawn will be higher; and (2) after the balloon is withdrawn, the paclitaxel concentration in the vessel will gradually decrease over time. The paclitaxel concentration in the vessel decreases faster within the first 24 h after the balloon is implanted and then slows down after 24 h. Analysis of the numerical simulation results shows that at the 10% stenosis rate, the optimal balloon dilation time should be 20 s. Under this dilation duration, the paclitaxel concentration in the blood vessel will drop below the toxic concentration within 24 h after balloon implantation and remain within the effective concentration range for the longest duration (about 6 weeks). Similarly, for the 30% vascular stenosis, the optimal balloon dilation time should be 60 s (with effective concentration duration for about 6 weeks). For the 50% vascular stenosis, the optimal balloon dilation time should be 80 s (with effective concentration duration for about 6 weeks).

## 4. Discussion and Conclusions

This study first modeled the paclitaxel-coated balloon and vessels chosen in the ex vivo experimental process. Finite element analysis and numerical simulation were then used to simulate the transfer process of paclitaxel from the balloon to the vessel. Some limitations exist. For example, the effect of blood flow was not considered during drug transfer. In reality, blood flow carries away some of the drug from the surface of the blood vessels, thus reducing the initial concentration. Additionally, the blood vessels regain their shape after balloon retraction, which was simplified and ignored in the simulation model. Since vascular deformation is primarily caused by the expansion force of the balloon, this study simplified the different layers of the vessel to homogeneous isotropic materials. This simplification has a relatively limited impact on vascular deformation.

Our study found that the transfer coefficient of the paclitaxel from the balloon surface to the vessel during the balloon dilation process is 1.28 × 10^−12^ m^2^/s. Next, finite element analysis of the dilation process was performed on three different stenosis rate (10%, 30%, and 50%) lesion vessel models, and the previously obtained transfer coefficient was used for numerical simulation of the paclitaxel transfer process from the balloon to the vessel, obtaining the paclitaxel content transferred to the three different stenosis rate vessel models at different dilation times. On this basis, the process of free diffusion of the paclitaxel in the vessel after the balloon was withdrawn was further numerically simulated. For a short balloon dilation time (20 s), the vascular paclitaxel concentration decreases to below 10 μg/g within a week, which is similar to the simulation result with a low-dose paclitaxel balloon obtained by Escuer et al. [30].

Our results show that after the balloon is withdrawn, the paclitaxel originally concentrated on the inner wall of the vessel will gradually diffuse evenly in the tunica media and tunica externa outer membrane of the vessel and in the axial direction at both ends of the lesion vessel. In addition, when the dilation time is within 120 s, the paclitaxel concentration in the vessel will gradually decrease over time to below the effective concentration. Finally, combining the clinical efficacy and safety criteria of using paclitaxel balloons, the study aimed to maximize the time during which intravascular paclitaxel concentrations remained above the effective concentration (0.047 μg/g) within 8 weeks, while ensuring that intravascular paclitaxel concentrations were below the toxic concentration (85.40 μg/g) within 24 h after balloon implantation. So, the conclusion was reached that the best dilation duration of the paclitaxel balloons should be 20 s, 60 s, and 80 s, respectively, when the stenosis rate of the lesion vessel is 10%, 30%, and 50%, providing a certain reference for clinicians to further optimize paclitaxel balloon implantation surgery plans.

## Figures and Tables

**Table 1:** Material property parameters of the vessel wall [25].

Material Property Parameters	Value
$\mu_{1}$	0.0002 MPa
$\alpha_{1}$	23.0320
$\mu_{2}$	0.0007 MPa
$\alpha_{2}$	0.9659
$\mu_{3}$	0.0007 MPa
$\alpha_{3}$	11.4302
$D_{1}$	11.4
$D_{2}$	0
$D_{3}$	0

**Table 2:** Material property parameters of the balloon.

Material Property Parameters	Value
Density	1256 kg/m^3^
Young’s Modulus	900 MPa
Poisson’s Ratio	0.3

**Table 3:** Comparison of simulation results and experimental results with different given transfer coefficients.

Transfer Coefficient (m^2^/s)	Paclitaxel Absorption Percentage in Vessels (%)		Total Error (%)
	60 s	120 s	
Experimental results	8.17	14.63	-
1.00 × 10^−12^	7.42	13.26	18.6
2.00 × 10^−12^	13.22	22.09	112.8
1.10 × 10^−12^	7.66	14.29	6.1
1.28 × 10^−12^	8.33	15.04	4.8

## Data Availability

The original contributions presented in this study are included in the article/supplementary material. Further inquiries can be directed to the corresponding author.

## Supplementary Materials

The following supporting information can be downloaded at: https://www.mdpi.com/article/10.3390/bioengineering13030301/s1, Table S1. Numerical simulation results of drug concentration (μg/g) changes over time after different balloon dilation times (stenosis rate is 10%); Table S2. Numerical simulation results of drug concentration (μg/g) changes over time after different balloon dilation times (stenosis rate is 30%); Table S3. Numerical simulation results of drug concentration (μg/g) changes over time after different balloon dilation times (stenosis rate is 50%).
