# Supplementary material for: Numerical Simulation and Optimization of Drug-Coated Balloon Inflation for Vascular Stenosis
Source: Bioengineering (Basel). 2026 Mar 5;13(3):301. doi: 10.3390/bioengineering13030301 (PMC13023489; doi:10.3390/bioengineering13030301)
Supplement: Supplementary file 1 [file bioengineering-13-00301-s001.zip › bioengineering-4122441-supplementary.pdf]

Supplementary Material

Table S1. Numerical simulation results of drug concentration (μg/g) changes over time after different balloon dilation times (stenosis rate is 10%)

Time After Balloon Withdrawal	Balloon Dilation Time					
	20s	40s	60s	80s	100s	120s
Initial	140.184	268.017	385.332	493.421	593.659	686.493
1 day	67.727	86.682	98.153	111.162	134.726	156.585
3 days	35.109	43.988	58.143	77.354	82.250	92.886
1 week	10.884	20.839	30.008	38.484	45.425	53.724
2 weeks	3.540	6.778	9.761	12.518	14.399	17.475
3 weeks	1.152	2.205	3.175	4.072	4.564	6.084
4 weeks	0.375	0.717	1.033	1.324	1.447	2.849
5 weeks	0.122	0.233	0.336	0.431	0.459	0.701
6 weeks	0.050	0.076	0.109	0.140	0.145	0.296
7 weeks	0.013	0.025	0.036	0.053	0.054	0.094
8 weeks	0.004	0.008	0.012	0.015	0.016	0.031

Table S2. Numerical simulation results of drug concentration (μg/g) changes over time after different balloon dilation times (stenosis rate is 30%)

Time After Balloon Withdrawal	Balloon Dilation Time					
	20s	40s	60s	80s	100s	120s
Initial	122.74	235.635	334.579	429.563	520.365	601.356
1 day	45.506	75.952	81.396	93.222	121.365	134.568
3 days	22.559	38.429	55.289	72.351	83.572	92.774
1 week	8.578	16.412	25.412	33.982	39.59	47.752
2 weeks	2.296	4.394	7.084	7.965	9.323	16.926
3 weeks	0.671	1.284	1.911	2.764	3.156	5.872
4 weeks	0.196	0.375	0.856	0.948	1.195	2.151
5 weeks	0.057	0.11	0.199	0.263	0.326	0.635
6 weeks	0.017	0.032	0.081	0.096	0.126	0.262
7 weeks	0.005	0.009	0.02	0.047	0.05	0.09
8 weeks	0.001	0.003	0.008	0.01	0.009	0.027

Table S3. Numerical simulation results of drug concentration (μg/g) changes over time after different balloon dilation times (stenosis rate is 50%)

**Time After Balloon Withdrawal**	**Balloon Dilation Time**					
	**20s**	**40s**	**60s**	**80s**	**100s**	**120s**
Initial	111.566	215.764	312.885	403.768	489.105	569.475
1 day	31.008	59.813	70.527	82.841	99.525	115.653
3 days	19.881	37.066	54.814	70.298	81.134	90.139
1 week	7.358	16.185	23.19	30.978	35.795	45.983
2 weeks	1.61	3.542	5.075	6.779	7.833	17.175
3 weeks	0.306	0.754	1.081	1.287	1.487	5.846
4 weeks	0.061	0.17	0.243	0.256	0.296	2.052
5 weeks	0.012	0.034	0.043	0.051	0.059	0.62
6 weeks	0.003	0.007	0.01	0.011	0.012	0.261
7 weeks	0.001	0.001	0.002	0.002	0.002	0.092
8 weeks	/	/	/	/	0.001	0.023
